# Seasonal Monitoring of Cardiovascular and Antiulcer Agents' Concentrations in Stream Waters Encompassing a Capital City

**DOI:** 10.1155/2013/753928

**Published:** 2012-11-03

**Authors:** Renáta Varga, Iván Somogyvári, Zsuzsanna Eke, Kornél Torkos

**Affiliations:** Joint Research and Training Laboratory on Separation Techniques, Eötvös Loránd University, Budapest 1117, Hungary

## Abstract

Nowadays monitoring pharmaceutical residues from surface waters is a widespread analytical task. Most of the studies are conducted from river waters or sewage treatment plants and mainly in Western Europe or North America. Such studies are seldom published from Eastern Europe, especially from stream waters, even though the prescription and consumption patterns of drugs as well as wastewater treatment procedures are very dissimilar. In Hungary the active substance of the most often prescribed drugs are cardiovascular and antiulcer agents. Hence in our study compounds belonging to these two groups were seasonally monitored in two main streams encompassing the Buda side of the Hungarian capital city and flowing into the Danube. To obtain data on the occurrence, fate, and seasonal variation of the compounds, samples were taken from altogether eleven points located near wastewater treatment plants and confluences. The results gave no identifiable pattern in the seasonal variation of concentrations but the contribution of the tributaries and wastewater treatment plants could be followed as expected. From the runoff corrected estuary concentrations the annual contribution of these streams to pharmaceutical pollution of the Danube could be estimated to be in excess of 1 kilogram for atenolol, famotidine, metoprolol, ranitidine, and sotalol.

## 1. Introduction

Pharmaceuticals are emerging contaminants in the environment. After digestion and excretion, due to nonefficient wastewater treatment procedures as well as the improper disposal of expired or nonused drugs they end up in surface waters. Regarding the facts that pharmaceuticals mean a continuous input in the environment and that they are designed to affect the human endocrine systems and additionally to be persistent it is worth to monitor their presence and variation in surface waters. Nowadays more and more surveys are published concerning the determination of pharmaceuticals from surface waters, especially from rivers and of course from sewage treatment plant influent and effluent samples to estimate the removal efficiency during the treatment processes [1–13]. It can be set out that most of these studies have been conducted in Western Europe and in North America but very little is known about the situation in Eastern Europe, even though the environmental concentrations may be very different due to differing patterns of usage, water consumption, and operation conditions of wastewater treatment. As far as we know similar study in Eastern Europe has been conducted only in Romania from Somes River [14–16] and in Hungary from Danube [17–19]. In these latter cases mainly the widely investigated nonsteroidal anti-inflammatories were monitored with GC-MS.

Once pharmaceuticals reach surface waters, they can be transformed mainly via biodegradation and photodegradation or they can adsorb onto suspended particles in the water. The concentrations in surface waters are very much dependent on the contribution of the wastewater flow to the receiving water flow and therefore on the dilution of the wastewater. Additionally, when a smaller water flow, such as a stream, flows into an exceedingly larger one the effect of dilution is even more significant. Regarding the aquatic organisms this secondary dilution has a positive effect because it means a less harmful milieu for them. But from the researchers' viewpoint it generates difficulties in carrying out an adequate environmental monitoring study: representative sampling, dilution corrections, and authoritative determination of low concentrations. Taking into account these elements of uncertainty we have designed an environmental loading study on pharmaceuticals in surface waters based on the two main streams encompassing the Buda-side of the capital city of Hungary. These streams both flow into the Danube, the second largest river in Europe. 

The aim of our study was (1) to obtain systematic, comprehensive, and measurement based data on the occurrence and fate of cardiovascular and anti-ulcer agents, in stream waters by seasonal sampling and (2) to estimate their environmental loading effect by calculating the streams' runoff data from their flow rate and cross-section at the estuaries. Furthermore the results of this study are expected to provide authoritative information for environmental scientists on real influent concentrations and on the contribution of wastewater treatment plants (WWTP) located in the suburban townships.

## 2. Material and Methods

### 2.1. Description of the Sampling Site Area and Sampling

Aranyhegyi Stream is a 23 km long water flow with a catchment area of 120 km^2^ which surrounds Buda from north. It has three main tributaries from the six townships of the catchment area with almost 41,000 inhabitants altogether. Along this stream one tertiary and two secondary WWTPs are located. Furthermore from one of the townships diffuse contamination can be expected due to the lack of a drainage system. 

Hosszúréti Stream is a 17 km long water flow with a catchment area of 114 km^2^ which surrounds Buda from south. It also has three main tributaries from the three townships of the catchment area with a population of more than 55,000 inhabitants altogether. Along this stream two tertiary and one secondary WWTPs can be found. Furthermore near a tract housing there is a damming to amend the quality of the stream water.

**Figure 1:**
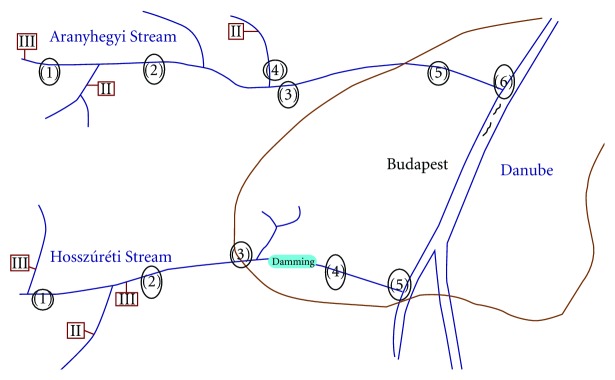
A schematic figure of the sampling area: numbers within circles indicate the sampling points; numbers within boxes indicate wastewater treatment plants (II—secondary, III—tertiary WWTP).

Four sampling campaigns were conducted in April, July, and October 2010 and in January 2011 (see Figure 1 and Table 1). With this seasonal sampling the effect of different weather conditions—such as air and water temperature, fall, and solar radiation—which can cause differences in the degree of dilution and photodegradation on the measured concentrations, could be followed. Along Aranyhegyi Stream six sample points were located: (1) after a tertiary WWTP, (2) after a secondary WWTP and the confluence of one of the main tributaries, (3) after another tributary confluence, in the area of the probable diffuse contamination, (4) precisely this sample was taken from the third main tributary after a secondary WWTP, but before the confluence to Aranyhegyi Stream, (5) where the stream enters into the area of Budapest, and finally (6) at the estuary. Along Hosszúréti Stream five sampling points were located: (1) after the first main confluence and a tertiary WWTP, but before a secondary one, (2) after a further tertiary and a secondary WWTP and the confluence of another main tributary, (3) before the confluence of the third main tributary, (4) after the water-quality amender damming, and finally (5) at the estuary (see Figure 1).

Owing to the fact that the streams are shallow and narrow all samples were taken from the mid-width of the streams and at least 10 cm under the surface. Samples were collected in a plastic vessel and at each point three parallels of 500 mL were exactly measured with a measuring cylinder into a brown amber glass bottle prerinsed with the actual stream water. All the samples were spiked with 500 *μ*L internal standard solution that contained all four internal standards at a concentration of 50 ng mL^−1^. The temperature of the samples was also measured on the spot. The samples were brought to the laboratory within a few hours, where their pH was also measured. The samples were stored at 4°C until processing but no longer than 48 h (see Table 1). 

To enable runoff calculations besides the flow rate the width and depth of the concreted stream basins were measured at the estuaries of both streams. The flow rate of the streams was determined as the average of the time needed for five equal sized floating sticks to take a predetermined distance. The runoff was calculated as the product of the cross-section and the flow rate. Based on the measured concentrations of each detected compound and the calculated runoff data annual environmental loading could be estimated.

### 2.2. Chemicals and Materials

All pharmaceutical standards were of high purity grade (>90%). Acebutolol, atenolol, betaxolol, carvedilol, cimetidine, esmolol, metoprolol, nifedipine, nizatidine, oxprenolol, propranolol, and sotalol were purchased from Sigma-Aldrich (Hungary). Atorvastatin-calcium, famotidine, lisinopril·2H_2_O, lovastatin, pantoprazole-sodium, ranitidine·HCl, ramipril, and simvastatin were from Wessling and Co. by courtesy. Atenolol-d_7_, enalapril-d_5_, and lansoprazole-d_4_ were purchased from CDN Isotopes (Quebec, Canada). Nimodipine and omeprazole were from Calbiochem (Darmstadt, Germany). Fluvastatin-sodium was from USP (Rockville, MD). Amlodipine besylate and enalapril maleate were from Richter Gedeon Co. by courtesy. Lansoprazole was from LGC Standards (Wesel, Germany).

Acetonitrile, methanol of HPLC gradient grade quality; acetone, n-hexane, and dichloromethane for gas chromatography; diethyl-ether and ethyl-acetate for chromatography were from Merck (Darmstadt, Germany). Water was deionized in our laboratory using a Millipore (Billerica, MA, USA) Milli-Q water purification system. Ammonium formate (cryst. extra pure, Ph Eur), ammonium acetate (cryst. extra pure, Ph Eur), formic acid (extra pure, Ph Eur), and acetic acid (extra pure, Ph Eur) were form Merck (Darmstadt, Germany). 25% aqueous NH_4_OH (analytical grade) was also from Merck (Darmstadt, Germany). Paper filters (3hw type) were purchased from Spektrum-3D (Hungary).

Standard and internal standard stock solutions of 1 mg/mL were prepared in methanol, with the exception of statin compounds (atorvastatin, fluvastatin, lovastatin, and simvastatin), which were prepared in acetonitrile because they proved to be degradable in methanol [17]. All stock solutions were stored at −18°C in a refrigerator for a maximum time of two months. Working and calibration solutions were prepared in 10% methanol in Millipore water and stored in the dark below 4°C.

### 2.3. Sample Preparation and Analytical Method

The details of sample pretreatment and the validated analytical method are discussed elsewhere [17] but briefly summarized below. 

All samples were filtered through paper filter before their pH was adjusted to 10 with 25% aqueous NH_4_OH. For solid phase extraction Oasis HLB cartridges (500 mg, 12 cc, Waters) were conditioned with 5 mL n-hexane, 5 mL acetone, 10 mL methanol, and then equilibrated with 10 mL Millipore water, pH adjusted to 10, with 25% aqueous NH_4_OH. 500 mL of samples were introduced to the cartridges through PTFE tubes at a flow rate of 3-4 mL min^−1^. After sample loading, the solid phase was washed with 2 mL of 5% methanol in 2% aqueous NH_4_OH. Cartridges were then dried for at least 10 min with air flow induced by the vacuum of the SPE manifold, and subsequently, the pharmaceuticals were eluted with 2 × 2.5 mL methanol. The effluents were evaporated to dryness by a gentle stream of nitrogen and reconstituted in 500 *μ*L of 10% methanol in Millipore water before injection.

Liquid chromatographic (LC) separations were carried out on an Agilent 1200 system (Agilent Technologies, Germany). Sample aliquots of 5 *μ*L were injected with needle wash (from flushport, acetonitrile-methanol 1 : 1 (v/v), 5 s) onto a Zorbax Eclipse Plus-C18 column (2.1 × 100 mm, 1.8 *μ*m) equipped with an in-line filter containing replacement frits (2 mm, 0.2 *μ*m). The column was kept at 50°C. Gradient elution was carried out at a flow rate of 250 *μ*Lmin^−1^ with 10 mM ammonium acetate, pH adjusted to 5 with acetic acid as eluent A and acetonitrile with 0.15% acetic acid as eluent B. The elution started with 10% eluent B and then the amount of it was linearly increased to 80% within 4 min, and within another 4 min to 100%. This eluent composition was held for 4 min, and then the percent of eluent B was immediately lowered back to 10%. Before the next injection, the system was allowed to equilibrate for 8 min generating a whole analytical run of 20 min.

The flow from the LC column was transferred to an Agilent 6460 Triple Quadrupole mass spectrometer (Agilent Technologies, Germany) equipped with an electrospray ionization source, supported by the new Agilent Jet Stream Technology. The temperature and the flow rate of the sheath gas were 350°C and 11 L min^−1^, respectively. Nitrogen was used as desolvation and nebulizer gas at a temperature of 350°C, a flow rate of 10 L min^−1^, and a pressure of 35 psi. The capillary voltage was 3500 V, while the nozzle voltage was 1000 V. The collision gas was also nitrogen. Positive ions were acquired in multiple reaction monitoring (MRM) mode. The compounds were grouped into two time segments. In the first one there were seven compounds with atenolol-d_7_ and cimetidine-d_3_ internal standards. In the second one were the other nineteen compounds with enalapril-d_5_ and lansoprazole-d_4_ internal standards. Dwell time of the first time segment was 50 ms, while that of the second one was 25 ms.

For quantitation a combination of matrix-matched and internal standard calibration was used. Matrix-matched calibration solutions were prepared by extraction of 100 mL Danube water samples applying the same sample pretreatment procedure as for the stream samples. After evaporation the samples were reconstituted in 500 *μ*L calibration solutions from 5 to 6000 ng mL^−1^ all containing the four internal standards at a concentration of 50 ng mL^−1^. Calibration curves were generated using linear regression analysis. For all compounds two MRM transitions were monitored: the more intensive was used for quantitation and the less intensive for confirmation. Other confirmation parameters were the ratio of the two MRM transitions and the retention time of the compounds (see Table 2). All three parallels of samples were injected two times and for further deductions the average of the six measured concentrations were used. Linearity, detection limit, and quantitation limit for each analyzed compound are summarized in Table 3.

## 3. Results and Discussion 

During the seasonal monitoring twenty-six pharmaceutical compounds (nineteen cardiovascular and seven anti-ulcer agents) were targeted and except the anti-ulcer substance cimetidine all of them were detected at least once. Five cardiovascular compounds (acebutolol, amlodipine, carvedilol, nifedipine, and simvastatin) were sporadically detected and three of them (acebutolol, amlodipine, and carvedilol) were only found in Aranyhegyi Stream. Some compounds showed a month characteristic appearance and could not be detected in each season: the cardiovascular acebutolol, amlodipine, and lovastatin were found only in October, the cardiovascular betaxolol, carvedilol, esmolol, and simvastatin were found in October and January, while the cardiovascular fluvastatin and the anti-ulcer lansoprazole and omeprazole were found in October and July. Furthermore the cardiovascular oxprenolol was only detected in January, enalapril was never detected in July, and with atorvastatin they were only found in January from Hosszúréti Stream, while lisinopril was found in July and January from Aranyhegyi Stream and in October and January from Hosszúréti Stream. The average, minimum and maximum concentrations of all the substances are summarized in Table 4. 

Ten compounds could be used to follow seasonal variations in concentrations as well as the WWTPs' contribution: four *β*-blockers, one ACE-inhibitor, one HMG-CoA reductase, three H_2_-antagonists, and one proton-pump inhibitor. For evaluating seasonal variations the measured concentrations of each compound were averaged over the sampling points in each season which is summarized in Table 5. Since no seasonal variation pattern of the concentrations can be seen, therefore it can be stated that the compounds' concentrations are more dependent on the substances physicochemical properties—that determine their persistence, removal efficiency, and transformation procedure in the environment—than on climatic or weather conditions. 

Overall the *β*-blocker metoprolol was detected at very high concentrations in both streams, and its highest concentration was measured in October. In Aranyhegyi Stream no big variations in the concentrations of atenolol, pantoprazole, and ramipril can be seen, while for famotidine, nizatidine, and sotalol an extremely lower concentration was detected in October compared to the other three campaigns. Extraordinarily higher concentration was detected for atorvastatin in April, for propranolol in October, and for ranitidine in April and January. Regarding Hosszúréti Stream all the samples were less contaminated, the measured concentrations were lower in general. For atenolol and nizatidine the concentrations were similar in each month, while for pantoprazole and propranolol an extremely lower concentration was detected in April. Extraordinarily higher concentrations were measured for famotidine in April and January, for ramipril in July, for ranitidine in January, and for sotalol in April.

To facilitate the evaluation of the contribution of WWTPs and tributaries the measured concentrations were averaged at each sampling point over the four months and summarized in Table 6. 

On the contrary to seasonal variations in this case a pattern could easily be observed. In Aranyhegyi Stream a continuous decline in the measured concentrations can be observed from the first to the third sampling point for each compound. This is due to the effective dilution from the tributaries in spite of the WWTPs' contribution to contamination. The fourth sampling point was located in one of the tributaries that has a remarkable contribution to the concentrations of almost all compounds, except atorvastatin and ramipril. Comparing the third sampling point with the fifth one four compounds' concentrations were almost unchanged (atenolol, atorvastatin, nizatidine, and ramipril), five compounds' concentrations decreased (famotidine, metoprolol, propranolol, ranitidine, and sotalol), while the concentration of pantoprazole increased. This latter observation indicates that for pantoprazole the contamination contribution of the tributary could not be diluted as effectively as for the other nine compounds. Even though between the last two sampling points there is not any expected contamination source some of the compounds (atorvastatin, famotidine, metoprolol, nizatidine, and sotalol) have higher concentration in the estuary sample than before. This can be explained only by a nonexpected contamination source such as illegal waste effluent or improper disposal of drugs. 

In Hosszúréti Stream the results of the first and the second sampling point reveal a remarkable and increasing contamination. Most probably the sources of this anticipated contamination are the tertiary and secondary WWTPs, the contribution of which cannot be compensated by the tributaries' dilution effect. In case of the third sampling point, however, only concentration decrease could be expected; nevertheless three compounds' concentration increased. Since the fourth sampling point was located after a damming a remarkable decline was expected in the concentrations. This expectation was met for almost all the compounds, but compared to the third sampling points results a little increase was observed for atenolol and sotalol and extremely (almost ten times) higher concentration was measured for ranitidine. These concentration increases can arise due to the activity of the microorganisms in the damming lake which reconvert the glucuronide conjugates of the pharmaceutical metabolites into the parent compound. The occurrence of such processes in surface waters is already known for various compounds [4, 20, 21]. For the estuary sample almost all compounds' concentrations decreased. The exception of famotidine can be explained only with a non-expected contamination source again. 

For authoritative environmental loading the runoff was calculated for both streams in each sampling campaign and the measured concentrations of each compound in the estuary samples were multiplied by those data and the annual contribution of pharmaceuticals to the Danube River pollution was estimated which is summarized in Table 7. 

Twenty-two compounds could be detected in the estuary samples altogether. In general the estimated annual environmental loading from the targeted pharmaceuticals does not reach one kilogram per year, but there are some compounds which exceed this limit. For both streams these are *β*-blockers (Aranyhegyi: metoprolol, propranolol, sotalol; Hosszúréti: atenolol, metoprolol, sotalol) and two H_2_-antagonists (famotidine and ranitidine). It is again really conspicuous that the *β*-blocker metoprolol causes the highest contamination and its estimated annual contribution to Danube's pollution exceeds 3 kilograms in each campaign from both streams; moreover the estimated annual loading from the Aranyhegyi Stream in October almost reaches 50 kilograms for that compound which cannot be effectively diluted neither in a high volume surface water as Danube. 

Therefore these estimations demonstrate that the usually measured ng L^−1^ concentrations of pharmaceuticals from surface waters cause a really high environmental loading annually which means remarkable environmental risk for aqueous living organisms and it should be amended with more effective wastewater treatment processes in Hungary as well.

## 4. Conclusions 

In our study the two main streams encompassing the Buda-side of the Hungarian capital city were sampled in four sampling campaigns at eleven points seasonally. Twenty-six cardiovascular and anti-ulcer agents, as the most prescribed drugs in Hungary, were targeted. From the measured data seasonal variation patterns could not be observed, which means that the climatic and weather conditions have less effect on the concentrations than the physicochemical properties of the pharmaceutical compounds. The eleven sampling points were chosen to facilitate the monitoring of the effect of the main tributaries and the nearby located WWTPs. In most cases these effects followed our expectations but in some cases non-expected contamination sources had to be supposed. The effect of a supposed water-quality amender damming was also investigated and in case of ranitidine the opposite effect was observed probably due to the glucuronide metabolite reconverting activity of microorganisms based on the estuary samples annual environmental loading was calculated and notable concentrations were estimated for *β*-blockers, namely, atenolol, metoprolol, propranolol, sotalol, and for H_2_-antagonists, namely famotidine and ranitidine. These estimations should be a warning for everybody: wastewater treatment processes should be ameliorated to protect aqueous living organisms.

Finally, it should be mentioned that the shown data in this paper are only a good indicator of the expected concentrations, but they could not be completely accurate due to some occasional variations during nonstudied days in the sampling points. More data should be collected in future months to study the tendency of the concentrations.

## Figures and Tables

**Table 1:** Sampling campaigns' characteristics.

Sampling campaign	Air temp. (°C)	Average water temp. (°C)	Water pH	Fall (mm/day)	Runoff (m^3^ s^−1^)	
					Aranyhegyi Stream	Hosszúréti Stream
April 2010	7	10.6	7.8–8.2	9	0.6628	0.7368
July 2010	27	25.3	7.8–8.2	0	0.2041	0.5904
October 2010	15	12.6	7.8–8.2	0.5	0.6297	0.4465
January 2011	0	4.3	7.8–8.2	7	0.8057	0.4200

**Table 2:** Compound-specific LC-MS/MS parameters of the investigated compounds.

Compounds	*R*_*t*_	TS	FragV	MRM1	CE1	MRM2	CE2	MRM ratio
Acebutolol	6.037	2	70	337.1 > 116	25	337.1 > 218	25	16.0
Amlodipine	6.946	2	100	409.1 > 238	10	409.1 > 294.1	10	55.4
Atenolol	1.629	1	130	267.1 > 144.9	25	267.1 > 190	20	45.6
Atenolol-d_7_	1.612	1	120	274.2 > 145	25	274.2 > 79.1	20	109.3
Atorvastatin	8.082	2	120	559.4 > 440.3	20	559.4 > 466.2	15	14.8
Betaxolol	6.711	2	70	308.1 > 116.1	20	308.1 > 161	20	23.9
Carvedilol	6.880	2	150	407.1 > 224.1	25	407.1 > 283	20	81.8
Cimetidine	1.597	1	90	253.1 > 95	30	253.1 > 159	10	90.7
Cimetidine-d_3_	1.598	1	70	256.1 > 162.1	10	256.1 > 95.1	25	88.4
Enalapril	6.650	2	140	377.2 > 234.2	15	377.2 > 303.2	15	28.4
Enalapril-d_5_	6.637	2	120	382.2 > 239.1	15	382.2 > 308.2	15	39.4
Esmolol	6.265	2	100	296.1 > 219	15	296.1 > 254.1	15	26.8
Famotidine	1.551	1	60	338.1 > 189	15	338.1 > 155	30	52.9
Fluvastatin	8.100	2	130	412.2 > 224	30	412.2 > 266.1	15	85.3
Lansoprazole	7.118	2	80	370.1 > 252.1	10	370.1 > 119.1	15	30.0
Lansoprazole-d_4_	7.107	2	90	374.1 > 252	5	374.1 > 123	20	36.7
Lisinopril	3.051	1	110	406.3 > 84.1	30	406.3 > 246.2	20	24.8
Lovastatin	9.729	2	50	405.3 > 199.1	10	405.3 > 285.1	5	90.7
Metoprolol	6.119	2	140	268.2 > 116.1	15	268.2 > 74.1	20	93.8
Nifedipine	7.712	2	70	347.1 > 315.1	0	347.1 > 254.1	15	56.3
Nimodipine	8.466	2	70	419.2 > 343.1	5	419.2 > 301.1	25	56.6
Nizatidine	1.896	1	100	332.1 > 58.1	30	332.1 > 155	15	40.9
Omeprazole	6.680	2	100	346.1 > 198	10	346.1 > 136.1	30	46.7
Oxprenolol	6.391	2	110	266.1 > 72.2	15	266.1 > 116.2	15	31.5
Pantoprazole	6.899	2	110	384.2 > 200.1	10	384.2 > 138.1	30	91.6
Propanolol	6.588	2	90	260.1 > 116.2	15	260.1 > 183.2	15	71.6
Ramipril	7.139	2	120	417.3 > 234.2	25	417.3 > 130.1	25	22.3
Ranitidine	2.374	1	90	315.2 > 176.1	15	315.2 > 130.1	25	60.9
Simvastatin	9.746	2	80	419.3 > 199.2	5	419.3 > 285.2	5	89.3
Sotalol	1.748	1	100	273.1 > 255	5	273.1 > 133	30	60.8

^*^R_t_: retention time (min); TS: time segment; FragV: Fragmentor voltage (V); MRM1: quantifier transition; CE1: collision energy for MRM1 (V); MRM2: qualifier transition; CE2: collision energy for MRM2 (V); MRM ratio: ratio of the two MRMs.

**Table 3:** Linearity, detection limit (DL), and quantitation limit (QL) for each analyzed compound.

Compounds	Linearity (*R* ^2^)	DL (ng L^−1^)	QL (ng L^−1^)
Acebutolol	0.9971	0.2	1
Amlodipine	0.9998	5	10
Atenolol	0.9997	0.2	1
Atorvastatin	0.9992	4	5
Betaxolol	0.9979	1	2
Carvedilol	0.9985	5	10
Cimetidine	0.9924	0.2	1
Enalapril	0.9999	0.2	1
Esmolol	0.9974	5	10
Famotidine	0.9949	1	2
Fluvastatin	0.9979	1	2
Lansoprazole	0.9996	4	10
Lisinopril	0.9985	—	—
Lovastatin	0.9996	3	10
Metoprolol	0.9966	0.2	1
Nifedipine	0.9989	5	15
Nimodipine	0.9991	5	10
Nizatidine	0.9824	1	2
Omeprazole	0.9993	1	2
Oxprenolol	0.9978	3	8
Pantoprazole	0.9952	2	4
Propranolol	0.9975	1	2
Ramipril	0.9997	0.5	2
Ranitidine	0.9841	0.5	2
Simvastatin	0.9989	3	10
Sotalol	0.9985	0.2	1

**Table 4:** Measured concentrations.

Compound	Concentrations (ng L^−1^)					
	Aranyhegyi Stream			Hosszúréti Stream		
	Min.	Max.	Average	Min.	Max.	Average
Acebutolol	16	261	98	—	—	—
Amlodipine	25	25	25	—	—	—
Atenolol	8	168	55	22	138	68
Atorvastatin	8	1102	114	12	125	33
Betaxolol	11	135	58	17	41	27
Carvedilol	12	58	41	—	—	—
Enalapril	1	42	8	2	49	13
Esmolol	<LOQ	121	41	<LOQ	40	18
Famotidine	12	782	153	11	528	150
Fluvastatin	11	150	56	2	9	5
Lansoprazole	<LOQ	10	7	<LOQ	650	87
Lisinopril	13	32	18	13	26	20
Lovastatin	<LOQ	11	10	10	12	11
Metoprolol	143	6944	1230	77	1150	525
Nifedipine	<LOQ	24	21	<LOQ	<LOQ	<LOQ
Nimodipine	<LOQ	<LOQ	<LOQ	<LOQ	<LOQ	<LOQ
Nizatidine	2	425	52	6	58	24
Omeprazole	6	9	8	6	6	6
Oxprenolol	<LOQ	34	14	<LOQ	15	14
Pantoprazole	4	166	32	4	40	15
Propranolol	2	189	36	7	65	29
Ramipril	3	56	14	4	33	13
Ranitidine	5	1451	257	15	478	167
Simvastatin	11	16	13	11	71	31
Sotalol	50	663	160	76	496	211

**Table 5:** Seasonal variations of the measured concentrations.

Compounds	Average concentrations (ng L^−1^)							
	Aranyhegyi Stream				Hosszúréti Stream			
	April	July	October	January	April	July	October	January
Atenolol	75	37	52	54	76	38	65	92
Atorvastatin	213	99	54	91	—	—	—	—
Famotidine	160	194	23	236	225	94	32	247
Metoprolol	257	871	3279	511	77	677	785	561
Nizatidine	57	88	10	52	30	13	11	41
Pantoprazole	33	13	20	61	4	14	28	12
Propranolol	5	21	95	24	7	32	46	30
Ramipril	14	17	8	16	8	26	8	8
Ranitidine	429	122	93	385	158	102	60	347
Sotalol	154	184	73	227	314	154	174	203

**Table 6:** Average concentrations at each sampling point.

Compounds	Average concentrations (ng L^−1^)										
	Aranyhegyi Stream						Hosszúréti Stream				
	1	2	3	4	5	6	1	2	3	4	5
Atenolol	84	74	21	47	19	19	19	89	69	70	59
Atorvastatin	461	95	22	2	19	26	—	—	—	—	—
Famotidine	452	173	76	50	36	45	47	198	254	83	112
Metoprolol	2534	1763	729	797	688	865	267	688	668	480	445
Nizatidine	21	9	11	205	12	15	10	38	27	20	14
Pantoprazole	20	17	6	61	37	5	7	16	21	8	7
Propranolol	65	42	28	31	23	25	20	36	31	23	19
Ramipril	30	12	4	4	4	5	8	11	14	4	2
Ranitidine	812	261	138	99	88	76	105	265	189	1480	103
Sotalol	377	163	77	178	65	80	104	274	254	261	164

**Table 7:** Estuary concentrations and runoff corrected annual environmental loading data (runoff data for calculation were summarized in Table 1).

Compounds	Aranyhegyi Stream								Hosszúréti Stream							
	Estuary concentrations (ng L^−1^)				Annual loading (kg year^−1^)				Estuary concentrations (ng L^−1^)				Annual loading (kg year^−1^)			
	April	July	October	January	April	July	October	January	April	July	October	January	April	July	October	January
Atenolol	31	19	—	25	0.65	0.12	—	0.64	65	22	56	92	1.51	0.41	0.79	1.22
Atorvastatin	19	51	12	20	0.40	0.33	0.24	0.51	—	—	<LOQ	52	—	—	—	0.69
Betaxolol	—	—	—	19	—	—	—	0.48	—	—	24	32	—	—	0.34	0.42
Carvedilol	—	—	45	11	—	—	0.89	0.28	—	—	—	—	—	—	—	—
Enalapril	3	6	<LOQ	4	0.06	0.04	—	0.10	—	—	—	17	—	—	—	0.23
Esmolol	—	—	—	—	—	—	—	—	—	—	25	<LOQ	—	—	0.35	—
Famotidine	59	—	12	107	1.23	—	0.24	2.72	51	—	47	350	1.19	—	0.66	4.64
Fluvastatin	—	—	—	—	—	—	—	—	—	—	<LOQ	—	—	—	—	—
Lansoprazole	—	<LOQ	<LOQ	—	—	—	—	—	—	—	<LOQ	—	—	—	—	—
Lisinopril	—	15	—	—	—	0.10	—	—	—	—	—	19	—	—	—	0.25
Lovastatin	—	—	11	—	—	—	0.22	—	—	—	—	—	—	—	—	—
Metoprolol	156	587	2486	229	3.26	3.78	49.37	5.82	—	293	823	665	—	5.46	11.59	8.81
Nimodipine	—	—	<LOQ	<LOQ	—	—	—	—	—	—	—	—	—	—	—	—
Nizatidine	31	25	—	5	0.65	0.16	—	0.13	9	—	7	39	0.21	—	0.10	0.52
Omeprazole	—	—	9	—	—	—	0.18	—	—	—	—	—	—	—	—	—
Oxprenolol	—	—	—	9	—	—	—	0.23	—	—	—	13	—	—	—	0.17
Pantoprazole	<LOQ	<LOQ	15	—	—	—	0.30	—	—	—	21	6	—	—	0.30	0.08
Propranolol	—	10	70	20	—	0.06	1.39	0.51	—	—	43	33	—	—	0.61	0.44
Ramipril	3	17	—	—	0.06	0.11	—	—	—	—	5	4	—	—	0.07	0.05
Ranitidine	146	29	—	128	3.05	0.19	—	3.25	74	—	63	275	1.72	—	0.89	3.64
Simvastatin	—	—	—	—	—	—	—	—	—	—	28	—	—	—	0.39	—
Sotalol	68	99	64	88	1.42	0.64	1.27	2.24	214	76	116	249	4.97	1.42	1.63	3.30
